# Conformational Altered p53 as an Early Marker of Oxidative Stress in Alzheimer's Disease

**DOI:** 10.1371/journal.pone.0029789

**Published:** 2012-01-05

**Authors:** Laura Buizza, Giovanna Cenini, Cristina Lanni, Giulia Ferrari-Toninelli, Chiara Prandelli, Stefano Govoni, Erica Buoso, Marco Racchi, Maria Barcikowska, Maria Styczynska, Aleksandra Szybinska, David Allan Butterfield, Maurizio Memo, Daniela Uberti

**Affiliations:** 1 Department of Biomedical Sciences and Biotechnologies, University of Brescia, Brescia, Italy; 2 Sanders-Brown Centre on Aging, University of Kentucky, Lexington, Kentucky, United States of America; 3 Department of Experimental and Applied Pharmacology, University of Pavia, Pavia, Italy; 4 Medical Research Centre Polish Academy of Science, Warszawa, Poland; 5 Laboratory of Neurodegeneration, International Institute of Molecular and Cell Biology, Warszawa, Poland; Virginia Commonwealth University, United States of America

## Abstract

In order to study oxidative stress in peripheral cells of Alzheimer's disease (AD) patients, immortalized lymphocytes derived from two peculiar cohorts of patients, referring to early onset AD (EOSAD) and subjects harboured AD related mutation (ADmut), were used. Oxidative stress was evaluated measuring i) the typical oxidative markers, such as HNE Michel adducts, 3 Nitro-Tyrosine residues and protein carbonyl on protein extracts, ii) and the antioxidant capacity, following the enzymatic kinetic of superoxide dismutase (SOD), glutathione peroxidase (GPx) and glutathione reductase (GRD). We found that the signs of oxidative stress, measured as oxidative marker levels, were evident only in ADmut but not in EOSAD patients. However, oxidative imbalance in EOSAD as well as ADmut lymphocytes was underlined by a reduced SOD activity and GRD activity in both pathological groups in comparison with cells derived from healthy subjects. Furthermore, a redox modulated p53 protein was found conformational altered in both EOSAD and ADmut B lymphocytes in comparison with control cells. This conformational altered p53 isoform, named “unfolded p53”, was recognized by the use of two specific conformational anti-p53 antibodies. Immunoprecipitation experiments, performed with the monoclonal antibodies PAb1620 (that recognizes p53wt) and PAb240 (that is direct towards unfolded p53), and followed by the immunoblotting with anti-4-hydroxynonenal (HNE) and anti- 3-nitrotyrosine (3NT) antibodies, showed a preferential increase of nitrated tyrosine residues in unfolded p53 isoform comparing to p53 wt protein, in both ADmut and EOSAD. In addition, a correlation between unfolded p53 and SOD activity was further found. Thus this study suggests that ROS/RNS contributed to change of p53 tertiary structure and that unfolded p53 can be considered as an early marker of oxidative imbalance in these patients.

## Introduction

Generation of reactive oxygen species (ROS), that are an inevitable by-product of cellular respiration, is believed to contribute substantially to the aging process [1]. Further increased ROS, as the consequence of pathological conditions as well as the exposure to endogenous and exogenous compounds, are, in turn, responsible for progressive decline in biological functions with time, and for higher predisposition to age-related disease, such as cancer, cardiovascular and neurodegenerative diseases [2], [3]. Persistent high levels of ROS/RNS can inflict direct damage to macromolecules, such as lipids, nucleic acids and proteins [4], impairing their functions, with a substantial physio-pathological impact [5]. The central nervous system (CNS) is very prone to oxidative imbalance because it is very rich of polyunsaturated fatty acids (PUFAs), has a high metabolic oxidative rate and high content of transient metals and ascorbate levels, which together act as pro-oxidant, but by contrast it possesses a relative paucity of antioxidant system compared with other organs [6].

Alzheimer's disease (AD) is the most frequent form of neurodegenerative disease associated with dementia in the elderly. Approximately 5% of AD is caused by mutations in the genes for either Amyloid precursor protein (APP) or some of the enzymes involved in its metabolism, Presenilin 1 and Presenilin 2 [7]. The remaining 95% are sporadic cases, whose causes are still unclear. Apart from the pathological hallmarks of the disease, which include accumulation of protein deposits in the brain as Aβ plaques and neurofibrillary tangles, AD brain exhibits constant evidence of ROS and RNS mediated injury [8]. Oxidative markers, such as 4-hydroxynonenal and malondyaldehyde, nitrotyrosine and protein carbonyls were found increased in *post mortem* AD brain [9]–[12]. Furthermore, different animal models of AD pathology, ei. Tg2576, APP23, APP/PS1 double knock-in, and triple Tg-AD, manifested features of lipid and protein oxidation at the early stage of their pathogenesis [13]–[15]. All these data support the basis of the oxidative stress hypothesis of AD.

Starting by the point of view that AD is a systemic disease, the oxidative imbalance, observed as oxidative damage in AD brain, may occur also in peripheral tissues of AD patients. Based on this concept, oxidative markers and the efficiency of antioxidant enzyme activity have been investigated in peripheral tissues of AD comparing them with those of healthy subjects [16]. The improvement in studying peripheral tissue, ei blood cells, is undoubtedly the easy accessibility of the biological sample on alive patient and the possibility to follow him in his history of illness. However, data in this contest are not so clear and are often contradictory.

Thus the aim of this study was to well characterize oxidative stress in AD taking advantage by the use of immortalized B lymphocytes derived from two peculiar cohorts of AD patients: patients harbouring AD-related mutation (ADmut) and sporadic AD, who developed the disease very early, and for this reason called Early Onset Sporadic Alzheimer's Disease (EOSAD); and comparing them with cells derived from healthy subjects.

Because our group previously demonstrated the expression of an anomalous tertiary structure of p53 protein in different peripheral cells derived from sporadic AD patients [17], [18] and many indications suggested p53 as a redox sensitive protein [19], [20], we also investigated whether a correlation between the expression of this conformationally altered p53 (unfolded p53) and oxidative profile in AD B lymphocytes exists.

## Results

### Oxidative profile in EOSAD and ADmut lymphocytes

Oxidative profile was evaluated measuring the expression of oxidative markers and the activity and levels of antioxidant enzymes in immortalized B lymphocytes derived from two peculiar cohorts of AD patients: sporadic cases with an early onset (EOSAD) and familial AD, named ADmut, because not all of them developed AD yet at the moment of blood withdrawal.

**Figure 1:**
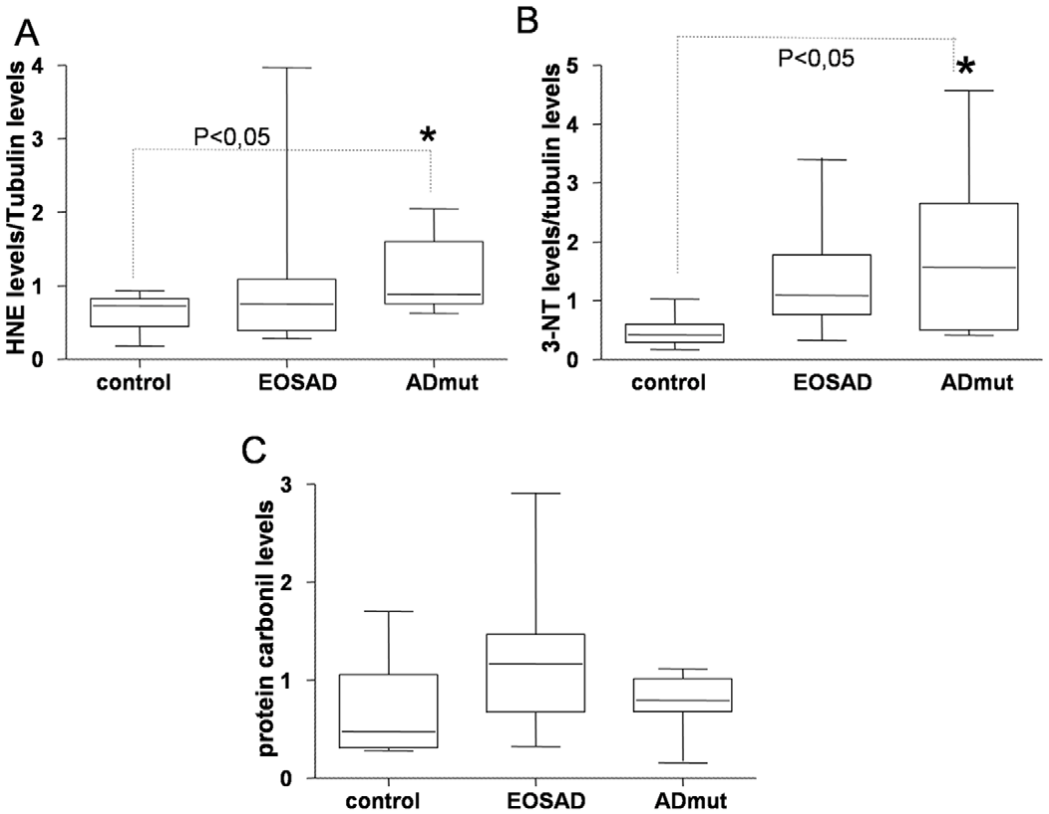
Oxidative profile in EOSAD, ADmut and control lymphocytes. Dot blot analysis on protein extracts derived from controls (n = 9), EOSAD (n = 9) and ADmut (n = 9) lymphocytes were performed using specific antibodies against oxidative stress markers: protein-bound HNE (**A**), 3NT (**B**) and PC (**C**). Tubulin expression was used to normalize the samples. * p<0,05 control vs ADmut.

4-hydroxy-2-nonenal (HNE), a product of lipid peroxidation [21]–[23] which by Michael addiction is able to bind proteins, 3-nitrotyrosine (3NT) [24], [25], a product of protein nitration and protein carbonyl (PC), derived from protein oxidation [26], [27], were measured using dot blot technique (Fig. 1). HNE adduct product levels and 3NT levels were found significantly enhanced in ADmut cells in comparison with control lymphocytes (mean value ± SEM: 1,12±0.49 vs 0,68±0,026; 1,8±0,12 vs 0,38±0,02 for HNE and 3NT respectively) (Fig. 1 A, B). In EOSAD samples, HNE adduct product (mean value ± SEM: 0,82±0,4) and 3NT levels (mean value ± SEM: 0,95±0,38) were no statistically different from controls. No statistically significant differences were found examining PC levels in ADmut and EOSAD in comparison with control cells (Fig. 1 C), probably due to the scatter of values (mean value ± SEM 0,48±0,045; 1,2±0,13; 0,78±0,07 for control EOSAD, and ADmut respectively). On the other hand, PC levels in ADmut were statistically significant in comparison with control by using the Bartlett's test for equal variances, but not with one way ANOVA and the Bonferroni tests (Fig. 1 C).

**Figure 2:**
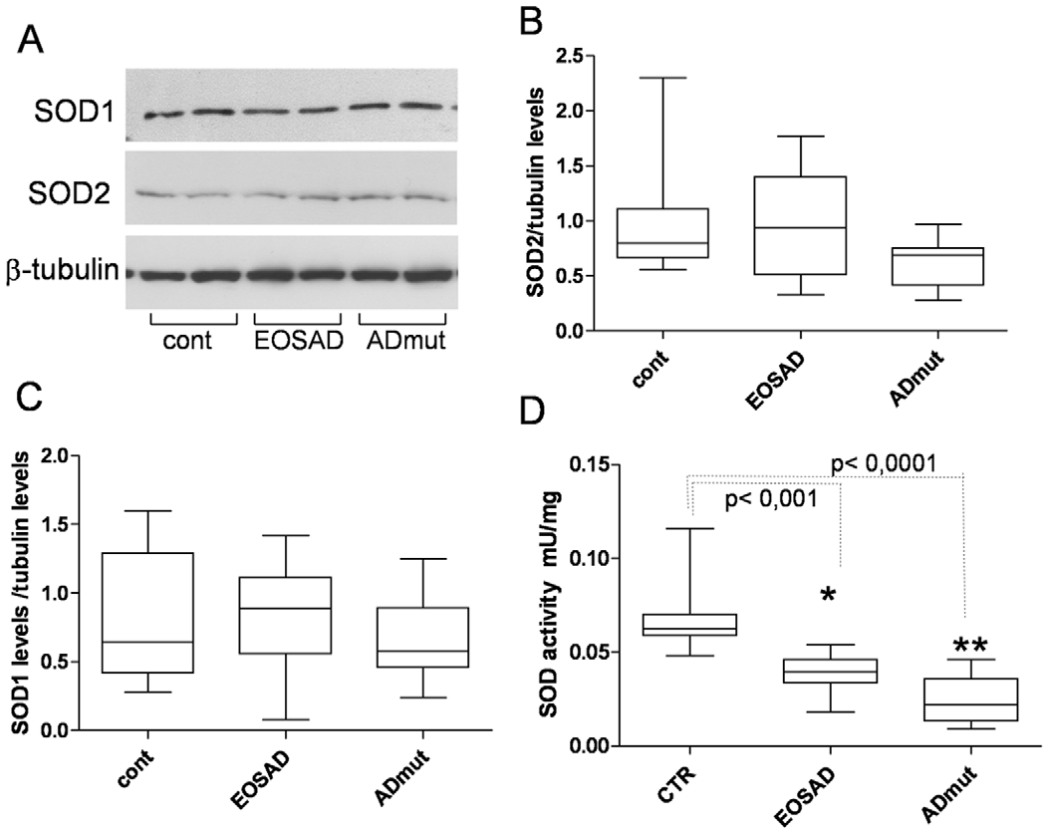
Expression of SOD1 and SOD2 protein levels and activity in ADmut, EOSAD and control lymphocytes. Protein extracts derived from immortalized lymphocytes of ADmut, EOSAD and healthy individuals were prepared as reported in method section. **A**) Western blot analysis carried out with monoclonal anti-SOD1 and anti-SOD2 antibodies on protein extracts derived from 2 controls, 2 ADmut, and 2 EOSAD. Tubulin expression was used to normalize the samples. **B**) and **C**) SOD1 and SOD2 levels of 9 controls, 9 ADmut and 9 EOSAD were measured using Scion Image program: quantitative analysis was expressed as intensity (optical density) of SOD1 or SOD2 bands over tubulin levels. **D**) enzymatic activity of superoxide dismutase (SOD) measured in controls (n = 9), EOSAD (n = 9) and ADmut (n = 9) lymphocytes using specific enzymatic assay (see method section).

Immortalized lymphocytes of the three groups were also processed for the measurement of SOD1 and SOD2 levels by western blot analysis and total SOD activity. In particular western blot analysis of protein extracts derived from control, ADmut and EOSAD lymphocytes were carried out with specific monoclonal anti-SOD1 and anti-SOD2 antibodies and then tubulin expression was used to normalize all samples. A representative experiment on 2 controls, 2 ADmut (PS1 H163R, APPT714A mutations) and 2 EOSAD is reported in Figure 2A. No difference in the expression of SOD1 and SOD2 was found in those samples. This trend was confirmed when the record of cases was increased, thus underlying a similar expression of SOD1 and SOD2 in ADmut, EOSAD and control lymphocytes (fig. 2B and C). SOD activity, measured as unit of enzyme able to inhibit epinephrine oxidation, was found lower in EOSAD and ADmut B lymphocytes in comparison with control cells, being any way lowest in ADmut samples (SOD mU/mg protein control: 0,062±0,006; EOSAD: 0.041^*^±0.002 ADmut: 0.025 ^**^±0,004 *p<0,001 vs control **p<0,0001 vs control) (fig. 2D).

**Figure 3:**
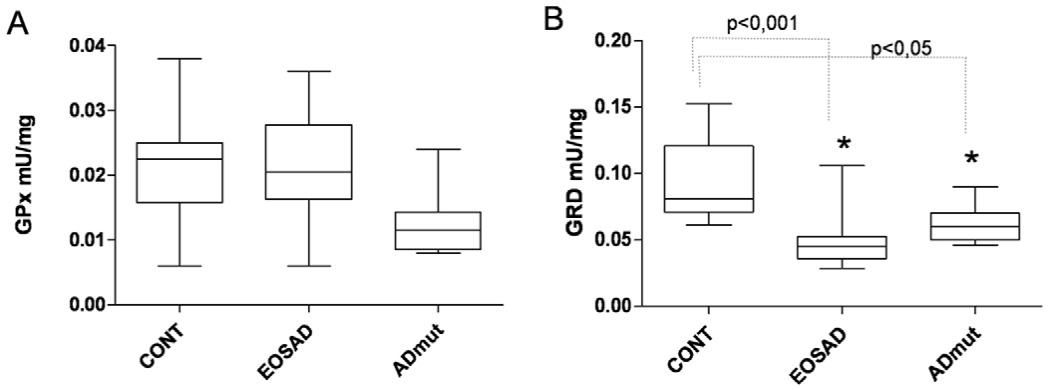
The enzymatic activity of glutathione peroxidase (GPX) and glutathione reductase (GRD). A) glutathione peroxidase (GPX) was measured in controls (n = 9), EOSAD (n = 9) and ADmut (n = 9) lymphocytes using specific assay (see method section). B) glutathione reductase (GRD) was measured in controls (n = 9), EOSAD (n = 9) and ADmut (n = 9) lymphocytes using specific assay (see method section).

The same samples were also processed for GPx and GRD activity (fig. 3). No difference was found in the expression of GPx activity (fig. 3 panel A) among the three groups of lymphocytes. The values of GPx activity were highly scattered, especially in the group of control and EOSAD, loosing statistically significant differences. At variance, GRD activity was lower in EOSAD (0,050±0,006 vs control 0,09±0,01, p<0,001) and ADmut immortalized lymphocytes (0,07±0,01 vs control 0,09±0,01, p<0,01) in comparison with control cells (fig. 3 panel B).

### p53 conformational state in EOSAD, ADmut and control lymphocytes

**Figure 4:**
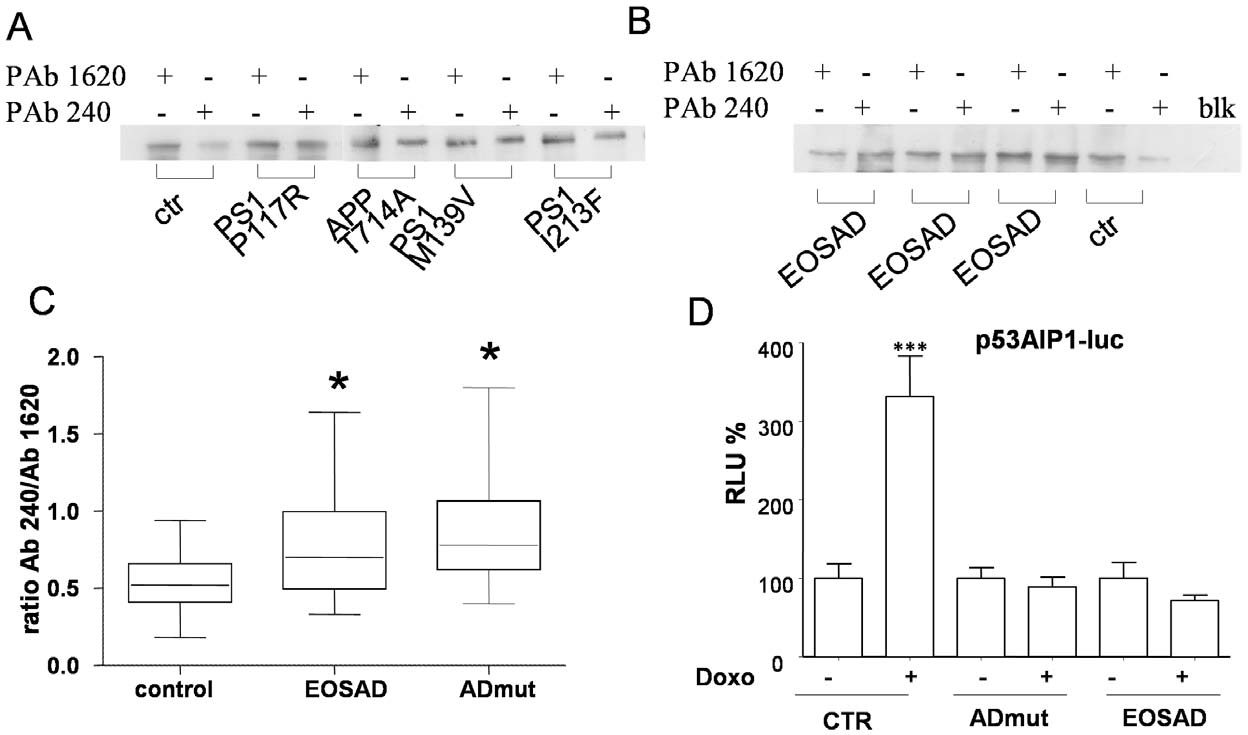
p53 conformational state in EOSAD, ADmut and control lymphocytes. Protein extracts derived from control, ADmut and EOSAD lymphocytes were immunoprecipitated by PAb240 (specific for p53 mutant isoform) and PAb1620 (specific for p53 wild-type isoform) antibodies. Immunoprecipitates were analysed by Western blot with the CM1 polyclonal anti-p53 antibody. (**A**) & (**B**) Representative blots of data from ADmut (n = 4), EOSAD (n = 3), and control (n = 2) lymphocytes. Immunoprecipitated antibodies were omitted in control (blk) samples (**C**) Ratio between the intensity of PAb240 and PAb1620 immunoreactive bands obtained from controls (n = 9), EOSAD (n = 9) and ADmut (n = 9) lymphocytes. Bars represent median value of the respective group. Data are expressed as mean ± SEM. * p<0,001. (**D**) Cells from control, EOSAD and ADmut subjects were transfected with p53AIP1-luc reporter construct and 18 h after transfection treated with doxorubicin (50 µM) for 24 h before luciferase activity was assayed. Luciferase activity was expressed as % of relative luminescence unit (RLU%) and compared to control values (cells without doxorubicin) assumed at 100%. Each bar represents the mean ± SD of six independent experiments. Statistical analysis was performed with Bonferroni multiple comparison test, with *** p<0.0001 vs CTR without doxorubicin.

Taking advantage by the use of two conformational specific anti-p53 antibodies, PAb1620 and PAb240, which discriminate folded vs. unfolded p53 tertiary structure [28], p53 conformational states were examined in immortalized lymphocytes derived from EOSAD, ADmut and healthy subjects. The experiments foresaw the immunoprecipitation with the two specific conformational antibodies, PAb1620 and PAb240 followed by immunoblotting with a rabbit polyclonal anti-p53 antibody. In particular PAb1620 binds to a denaturation-sensitive epitope within the DNA-binding surface [29], whereas PAb240 recognizes a primary epitope that is cryptic in the wild type conformation and becomes exposed when the protein changes its conformation towards an unfolded phenotype [30]. As showed in representative immunoprecipitation experiments, lymphocytes derived from healthy subjects expressed an intense band related to wild-type p53, as demonstrated by the reactivity with PAb1620, while immunoreactivity to PAb240 (unfolded p53) was very low. Three representative EOSAD and four ADmut cells (PS1P117R, APPT714A, PS1I123F and PS1M139V) expressed, besides the PAb1620-positive p53 isoform, a higher immunoreactivity to PAb240 (fig. 4 panel A, B). When an increased number of EOSAD (n = 9), ADmut (n = 9) and control (n = 9) cell lines was tested for immunoprecipitation experiments, two well separated p53 phenotype patterns were identified. The ratio of PAb240/PAb1620 was statistically higher in EOSAD and ADmut in comparison with control cells (Fig. 4 C). Furthermore, to test whether altered p53 conformation, found in EOSAD and ADmut lymphocytes, compromised p53 functionality, p53 transcriptional activity was evaluated by luciferase assay of the p53AIP1-luc apoptotic promoter [31]. The experiments were performed on cells derived from 1 control, 1 EOSAD and 3 ADmut (PS1H163R, PS2 Q228L and PS1M139V). Cells were transiently transfected with the p53AIP1-luc reporter plasmid and 18 hrs later treated with 50 µM doxorubicin, a cytotoxic agent able to induce DNA damage and apoptosis in a p53-dependent manner [32]. As shown in Figure 4 panel D, p53AIP1-luciferase activity was induced by doxorubicin treatment in cells from control subject, whereas it was significantly impaired in EOSAD and ADmut cells.

**Figure 5:**
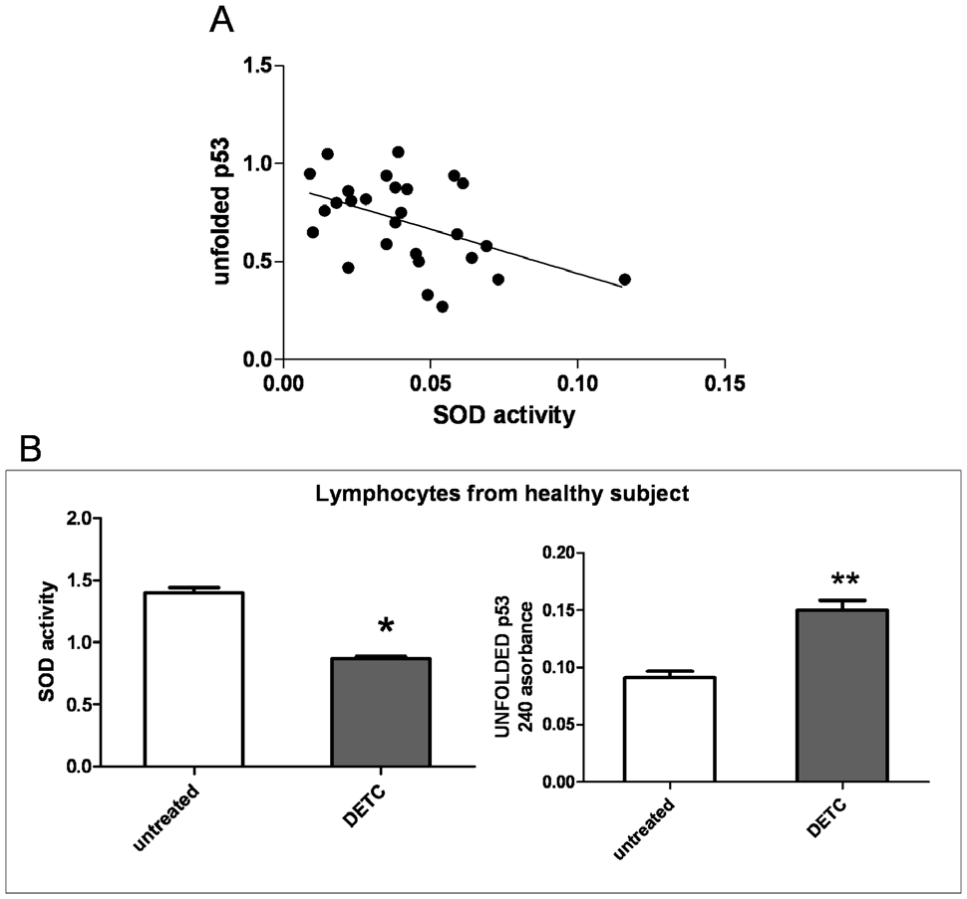
(A)correlative analysis between SOD activity and unfolded p53 on all samples (control, EOSAD and FAD) considered in this study. The equation of linear regression is y = −5,518x+0,1973 r^2^ 0,2321 p = 0,01. (**B**) lymphocytes derived from a healthy subject were exposed to SOD inhibitor DETC (5 Mm) and 24 h later they were processed for SOD enzyme activity and the expression of PAb 240 -positive p53 isoform by using ELISA assay. Data are expressed as mean ± SEM of three different experiments, performed in triplicate. Statistical analysis was performed with t test with * p<0,001, ** p<0,001 vs untreated samples.

It is noteworthy that the value of unfolded p53 was well correlated with SOD activity. Lower values of SOD were associated with higher value of unfolded p53 in both pathological and control cells (regression equation: y = −518 x+0,1973; r^2^ 0,2321; p = 0,01) (fig. 5, A). At variance, the decrease of GR activity did not correlate with unfolded p53 (data not shown). It is noteworthy that when a lymphocyte line derived from healthy subjects was exposed for 24 h with the SOD inhibitor, diethyldithiocarbamic acid (DETC) at the concentration of 5 mM, a 50% decrease of SOD activity and a comparable increase of PAb240 absorbance, indicative of unfolded p53 isoform, were observed, suggesting a strictly correlation between SOD activity and p53 conformational state (fig. 5 B).

### Oxidative modulation of p53 molecule in EOSAD and ADmut lymphocytes

**Figure 6:**
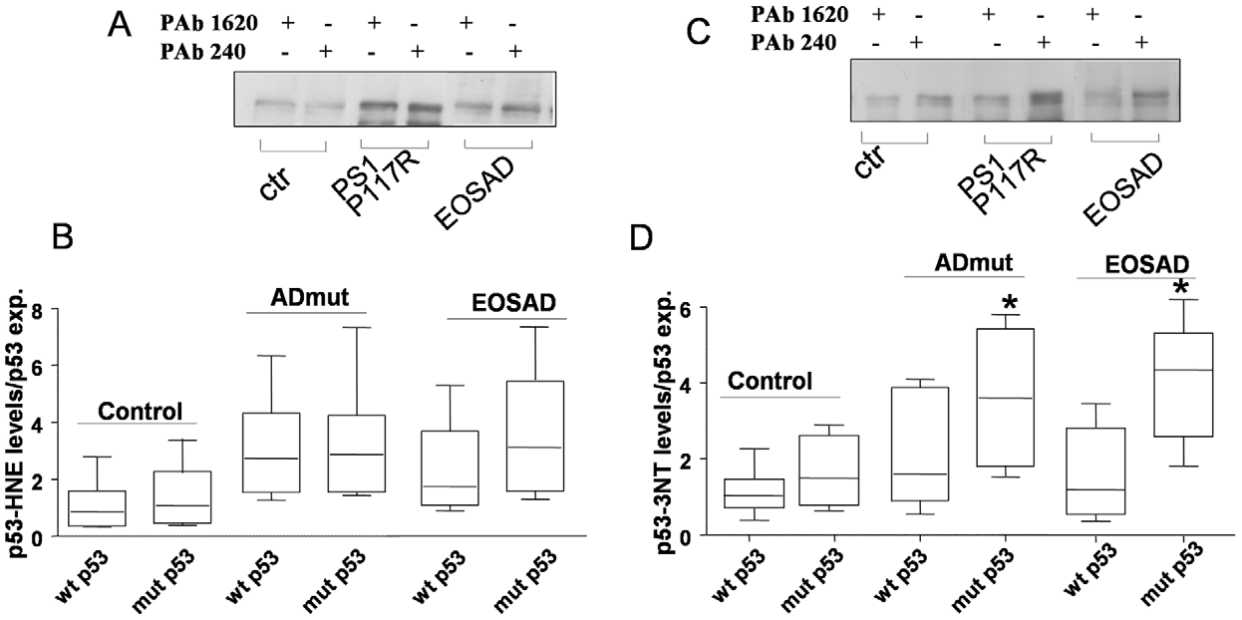
Oxidated/nitrated wild-type and mutant p53 in EOSAD, ADmut, and control lymphocytes. Representative blot of data obtained from PS1P117R mut (n = 1), EOSAD (n = 1) and control (n = 1) lymphocytes. Equal amount of protein from control, ADmut and EOSAD lymphocytes were immunoprecipitated with conformation specific anti-p53 antibodies, and immunoprecipitates were analyzed for HNE (**A**) or 3NT (**B**) immunoreactivity by Western blotting. (**C**) Graphical analysis of HNE-p53 band intensities of controls (n = 5), EOSAD (n = 5), and ADmut (n = 6). p53 expression was used to normalize the intensity of each band. Bars represent median value of the respective group. Data are expressed as mean ± SEM. (**D**) Graphical analysis of 3NT-p53 band intensities of controls (n = 5), EOSAD (n = 5) and ADmut (n = 6). p53 expression was used to normalize the intensity of each band. Bars represent median value of the respective group. Data are expressed as mean ± SEM. * p<0,01.

**Figure 7:**
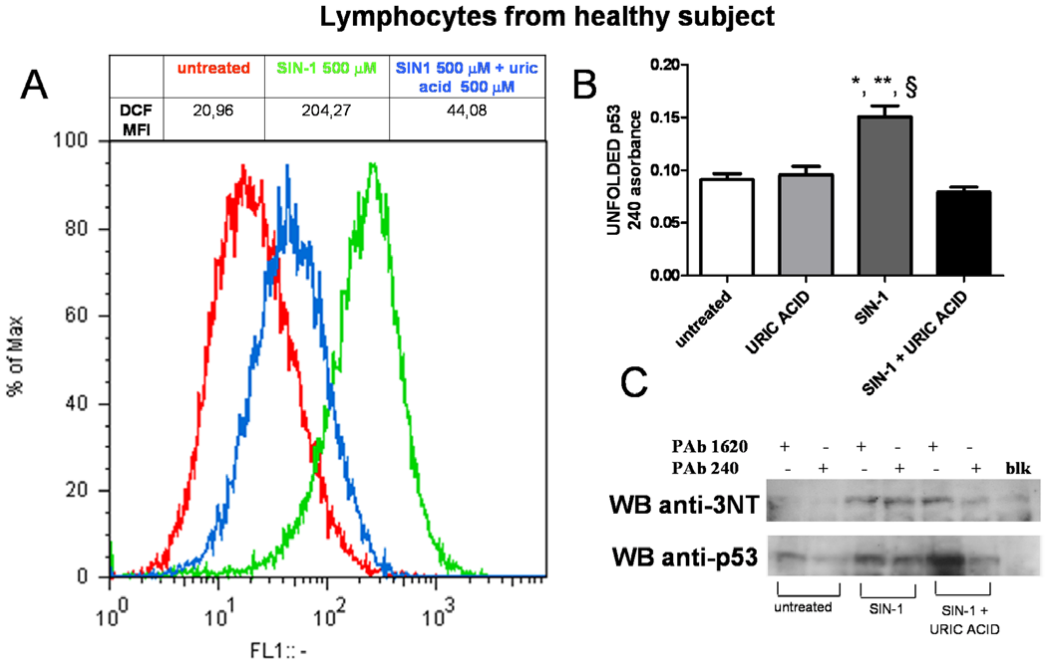
Effects of peroxynitrite compound SIN-1 on p53 conformation. Lymphocytes derive from a healthy subject were exposed to 500 µM SIN-1 in the presence or absence of uric acid at the same concentration. In particular SIN-1 was added 30 min after uric acid addition and incubated for the next 4 hours. Cells were then processed for (**A**) RNS generation study by FACS analysis measuring DCF fluorescence; (**B**) Pab 240 positive p53 isoform (unfolded p53) measured by ELISA assay, and (**C**) the degree of p53 nitration on tyrosine residues investigated by immunoprecipitation experiment with the two conformational specific antibodies (PAb 1620 and pab 240) followed by immunoblottin, with anti-rabbit-anti-3NT or anti-goat anti-p53 (R19).

Besides germ line mutations, conformational changes in p53 protein can occur following post-transcriptional modifications [28], [33], [34]. Numerous studies reported that cysteine oxidation inside the DNA binding domain can affect the p53 conformational status [20], [35], [36]. Oxidative modulation of p53 was assessed by immunoprecipitating the protein with conformational specific antibodies, and then blotting the membranes with anti-HNE and anti-3NT antibodies. The data were normalized with the levels of wild type and unfolded p53 respectively. Figure 6A shows a representative experiment performed on one control, one ADmut (PS1 P117R mutation) and one EOSAD. PS1 P117R mutated lymphocytes showed increased levels of p53-bound HNE in comparison with control. In particular HNE Michael adducts were found in both wild type and unfolded p53 phenotypes. However, when more samples were processed, no statistically significant differences among ADmut, EOSAD and control lymphocytes were observed in p53-bound HNE levels (mean value ± SEM; control cells: p53wt 0,60±0,12, p53 unfolded 0,82±0,09; ADmut lymphocytes p53wt 2,7±1,68 p53 unfolded 2,9±1,01; EOSAD p53wt 1,28±0,85 p53 unfolded 2,8±1,05) (Fig. 6B). The measurement of the amount of nitrated tyrosine residues on p53 molecule showed a more intense band in the samples immunoprecipitated with PAb240 antibody in comparison with those immunoprecipitated with PAb1620 (Fig. 6 C). In both PS1P117R mutated and EOSAD cells unfolded p53 was more nitrated than unfolded p53 of control cells (Fig. 6C). This pattern was confirmed also in the other ADmut, EOSAD and control samples examined. Figure 6D depicted the quantitative analysis of p53-3NT levels over corresponding wild type and unfolded p53 levels of 5 controls, 6 ADmut and 5 EOSAD cells. The expression of p53-3NT was found statistically increased both in EOSAD and ADmut lymphocytes in comparison with control cells, when the protein was in an unfolded conformation, recognized by PAb240 antibody (mean value ± SEM: control cells: p53wt 0,86±0,18, p53 unfolded 1,01±0,92; ADmut lymphocytes p53wt 1,28±0,60 p53 unfolded 3,82±0,64; EOSAD p53wt 0,88±0,35 p53 unfolded 4,00±0,95) (Fig. 6D). To give more insight on the effects of tyrosine nitration on p53 tertiary structure, lymphocytes derived from one healthy subject were exposed to a peroxynitrite-generating compound, 3-morpholinosydnonimine hydrochloride (SIN-1), at the concentration of 500 µM in the presence or absence of uric acid (500 µM), a peroxynitrite scavenger [37]. Four hours later, FACS analysis demonstrated increased RNS generation induced by SIN-1, that was prevented by the co-exposure with uric acid, as shown by the shift of DCF fluorescence (fig. 7A). Peroxynitrite-generating compound increased also PAb240 absorbance in ELISA assay and this effect was reverted by uric acid (fig. 7B). Finally, immunoprecipitation experiments were performed using the two specific conformational anti-p53 antibodies, PAb1620 and PAb240, followed by immunoblotting with anti-3NT antibody or anti-p53 antibody. As shown in figure 7C, SIN-1-induced tyrosine nitration was responsible of p53 conformational changes towards an unfolded phenotype. In particular, immunoprecipitated samples derived from cells treated with SIN-1 showed, in addition to a wt p53 isoform immunoreactive to PA1620, an intense PAb240 positive band. While untreated cells especially expressed wt p53 (PAb1620 positive p53 isoform). The co-exposure of SIN1 with uric acid prevented the increased expression of unfolded p53 immunoreactive to PAb240 (fig. 7C), even if the wt-p53 expression was anyway intense, more than untreated cells. Interestingly, SIN1 induced a high enhance of p53-3NT expression in both wt and unfolded p53 conformation in comparison with untreated. Uric acid co-treatment expressed high levels of wt p53-3NT isoform, due probably to the high immunoprecipitated PAb1620 positive p53 isoform. At variance unfolded p53-3NT isoform was very low when SIN-1 was co-added with uric acid (fig. 7C). These data confirmed that nitration on tyrosine residues of p53 molecule is responsible of protein conformational changes towards an unfolded phenotype.

## Discussion

In this study we demonstrated a correlation between oxidative profile imbalance and the expression of a conformationally altered p53 isoform in immortalized lymphocytes derived from two peculiar cohorts of AD patients: sporadic cases with an early onset (EOSAD) and a group of subjects harbouring AD mutations. This last group was called ADmut, because only some of the patients expressed clinical signs of AD at the time of blood withdrawal. An advantage of examining these ADmut subjects is that every alteration, found in them, can be associated with the pathogenesis of the disease. Furthermore, EOSAD subjects, although they did not harbour the most known AD-mutations, represented a very homogeneous group. Oxidative profile was studied using two parameters: 1) the measurement of typical oxidative stress markers, such as HNE Michael adducts, as product of lipoperoxidation, 3NT, derived from nitration to tyrosine residues and PC, as a direct protein oxidation; 2) the quantification of antioxidant capacity, carried out as the activity of SOD, GPx and GRD enzymes.

Increased oxidative markers, such as HNE and 3-NT, were observed in ADmut, but not in EOSAD lymphocytes. These data are apparently in contrast with our previous observations on sporadic AD brains. Increased oxidative markers were, in fact, found in hippocampus and inferior parietal lobe of both sporadic AD and MCI brains in comparison with control [38]–[40]. The discrepancy between data reported in brain and peripheral cells of EOSAD could be due to the higher susceptibility of CNS to free radical damage, because brain has a high oxygen consumption rate, abundant lipid content and a relative paucity of antioxidant enzymes compared with other tissues [41]. It is presumable to assume that, in periphery, oxidative stress takes more time to develop because probably more efficiently endogenous and exogenous (for example from the diet) antioxidants influence and modulate ROS/RNS (reactive nitrogen species) generation. Consistent with our observations, other studies failed to find robust evidence of increased peripheral oxidative stress in sporadic AD (SAD). Cecchi et al [42] have demonstrated that lipoperoxidation in lymphoblast from patients affected by SAD was virtually indistinguishable from the basal values of normal controls. On the contrary more reproducible results were obtained on peripheral cells derived from patients affected by familial AD. In fact lymphoblasts derived from patients harbouring APP and PS1 mutation showed increased expression of oxidative markers [43].

At variance, an oxidative imbalance in EOSAD was demonstrated by a reduced antioxidant capacity. SOD activity was decreased in both ADmut and EOSAD when compared with control, although the expression of SOD1 and SOD2 protein did not change in the two pathological groups in comparison with control. The fact that the expression of SOD enzymes does not always correlate with their activity has been already reported in AD animal models and patients [14], [44]. It is noteworthy that SOD enzyme is itself a target of oxidation, affecting its activity. At this regard, chronic treatment with low concentration of 5 µM H_2_O_2_ reduced SOD2 activity in HeLa cells. This event was prevented by ascorbic acid and N-acetylcysteine, suggesting that also SOD2 can be affected by the same ROS it neutralized [45]. Recently Bosco et al [46] demonstrated that oxidation of wt SOD1 protein induced conformational changes and decreased the efficiency of this enzyme activity. Also GRD, but not GPX, activity was decreased in EOSAD and ADmut lymphocytes.

EOSAD and ADmut further expressed a detectable amount of unfolded p53 isoform. p53 conformational state was measured with immunoprecipitation experiments using two specific conformational antibodies, that recognized the wild type (PAb1620) and altered (PAb240) tertiary structure. Unfolded p53, measured as the ratio between PAb1620 and PAb240 immunoreactivity was found statistically enhanced in immortalized lymphocytes derived from EOSAD in comparison with control subjects. In addition, also the majority of ADmut subjects, harbouring different mutations in the APP, PS1, and PS2 genes, who were diagnosed AD at the time of blood withdrawal, showed increased unfolded p53 in comparison with control subjects. Lymphoblastoid cell lines obtained by infecting peripheral blood mononuclear cells with the Epstein Barr virus, have been documented to retain the cellular response of freshly cells that they had at the moment of withdrawal [47], [48]. Thus, some observations on these familiar cases can be made. PS2 Q228L mutation was found in a subject with MCI, with a MMSE score of 28 that remained unaltered after 2 years. PS2Q228L unfolded p53, measured as a ratio between PAb240/PAb1620, has a value of 1,54 vs. mean control of about 0,5±0,08. One PS1 mutation, PS1 S170F, is a 9 years old child, whose parent, harbouring the same mutation, is affected by the disease. The child, of course, does not have any signs of pathology, however child's lymphocytes expressed an unfolded p53 value of 0,80 and parent's of 1,05. These results were consistent with those obtained by independent scientific groups. We previously found higher expression of PAb240 positive p53 isoform in fresh mononuclear blood cells derived from sporadic AD patients and MCI patients, who converted in AD followed two-year study, in comparison with control subjects [18], [49]. Zhou and Jia [50] demonstrated a p53-mediated G1/S checkpoint dysfunction in lymphocytes from sporadic AD, due to p53 conformational changes that affected its tertiary structure. Furthermore, Serrano et al., [51] demonstrated a significant increase of unfolded p53 in older AD transgenic mice when compared with younger APPswe/PS1A246E animals and wild-type counterparts of the same age. These latter data suggest also a correlation between unfolded p53 and aging. At this regards we indeed found a linear correlation between aging and unfolded p53 in both control subjects and AD patients, with the highest values of unfolded p53 in AD at each age examined [49].

A role of oxidative imbalance in p53 conformational changes was suggested by the inverse correlation between unfolded p53 and SOD activity: a decrease of SOD activity was associated with an increase of unfolded p53 expression. Furthermore, the SOD inhibitor DETC, added to control lymphocytes for 24 h, was able to recapitulate AD phenotype, inducing p53 conformational changes towards a unfolded phenotype.

p53 belongs to a growing list of transcriptional factors, which are subjected to redox modulation [52]–[54]. Reactive oxygen intermediates (ROS) play at least two distinct roles in the p53 pathway. First, they are important activators of p53 through their capacity to induce DNA strand break [17], [55], [56]. Second, they regulate the DNA-binding activity of p53 by modulating the redox state of a critical set of cysteines in the DNA-binding domain, which in turn induces conformational changes [33], [34], [36]. The duration and the degree of ROS signalling can influence one or the other event.

When we measured the degree of HNE oxidation and nitration of wild type and unfolded p53 isoforms, we preferentially found a dramatic increase of p53-3NT in mutant conformation, both in EOSAD and ADmut in comparison with control lymphocytes. The effects of tyrosine nitration on p53 conformational state was also demonstrated by the using of peroxynitrite-generating compound SIN1. SIN1 induced the expression of PAb240 positive p53-3NT isoform, while in untreated cells this peculiar isoform was virtually absent. However also the p53 wt conformation was found nitrated in a higher extent in cells exposed to SIN-1 than in untreated cells. This is plausible since the p53 molecule contains 15 tyrosine residues, and 11 of them are inside the highly flexible DNA binding domain. In accordance with our finding, synthetic nitric oxide donors (such as S-nitro-N-acetyl-penicilamine and S-nitrosoglutathione) have been shown to induce a conformational switch of wild-type p53 to the unfolded form, with loss of DNA-binding activity *in vitro* [57]. It is well recognized that the nitration of tyrosine at the 3-position sterically hinders the phosphorylation and also may change the structure of proteins, thus making protein dysfunctional [58]–[60].

It is noteworthy that the young patient, who harboured a PS1 S170F mutation, expressed high levels of unfolded p53 and unfolded nitrated p53, but low levels of oxidative markers comparable to those found in control lymphocytes. Therefore, we suggest that oxidation of p53 protein may be an early event in establishing oxidative stress in periphery. In summary, we demonstrated that, although in EOSAD patients oxidative stress is not detectable in periphery when measured as oxidative markers, the sign of an unbalanced redox state in these patients is demonstrated by the effects that reactive nitrogen species (RNS) have on p53 protein.

## Materials and Methods

### Subjects

Alzheimer's disease patients and healthy subjects were enrolled in the Department of Neurology, MSWiA Hospital, Warsaw, Poland. The protocol of the study was approved by the Bioethical Committee for Studies on Human Subjects at the Central Clinical Hospital MSWiA in Warsaw, and is in compliance with the National and European Union legislation and the Code of Ethical Principles for Medical Research Involving Human Subjects of the World Medical Association. Approval No 68/2008 and a written consent was obtained from all subjects or, where appropriate, their caregivers. The demographic and clinical informations as well the mutations are reported in Table 1 and 2. Control subjects were individuals with no clinical signs of neurological or psychiatric diseases. All these subjects were examined by a senior neurologist or geriatrician and diagnosis of dementia was made according to DSM-IV and the NINCDS-ADRDA criteria. Dementia was diagnosed based upon interview, objective and neurological examination, cognitive evaluation, laboratory and radiological (CT Scan) investigation. Cognitive status was quantified using the Mini Mental State Examination (MMSE). AD patients represented both familial (ADmut) and sporadic forms of the disease. ADmut patients were carrying mutations in Presenilin 1 (PS1), Presenilin 2 (PS2) and Amyloid Precursor Protein (APP) genes and showed classical early onset of the disease (before their 50th). Patient with PS2 Q228L mutation was diagnosed as having mild cognitive impairment (MCI) and developed no AD features so far (5 years after first examination). In ADmut group there were also a patient with PS1 S170F mutation which caused very severe course of disease with the onset as early as at 29 years, and her 9 years old healthy child. Because these groups of subjects harboring AD mutations were not all affected by AD, they were named ADmut. On the other hand, patients with sporadic AD were not typical SAD cases. Despite they bear no mutations in PS1 and PS2 and APP genes, onset of the disease was at early 50th or even below comparing to the “classical” SADs with the onset over 65 years of age. Majority of those patients were also not bearing ApoE4 allele which is considered to be an AD risk factor (data not shown). We called that group an Early Onset Sporadic Alzheimer's Disease (EOSAD) patients.

**Table 1:** Demographic and clinical characteristic of the subjects enrolled in the study.

	EOSAD	ADmut	CTRL
**N (M;F)**	9 (5; 5)	9 (5; 4)	9 (6; 3)
**mean age ± SD**	64±5	44±9	65±2
**Age of onset**	54±5	42±8	-
**MMSE**	12,4±9	11,2±8,2	27,8±2,9

Abbreviations: EOSAD, early onset sporadic AD; ADmut, familial AD; CTR, control; M, male; F, female; LOI, length of illness; MMSE, Mini Mental State Examination; N, number of individuals.

Values are expressed as mean ± SD.

**Table 2:** Type of mutations, age of onset, and age of ADmut patients.

Mutations	Age of onset	Age
PS1 H163R	50	59
PS2 Q228L	60	67
PS1 M139V	40	46
APP T714A	44	51
PS1 P117R	36	42
PS1 I213F	33	41
PS1 S170F	29	34
PS1 S170F		9
PS1 L153V	<40	41

Abbreviations: ADmut, familial AD, PS1, presenilin 1, PS2, presenilin 2, APP, Amyloid Precursor Protein.

### Cell cultures

Immortalized B lymphocytes derived from enrolled patients (see above Bioethical Committee and confirmation of written inform consent) were used [61]. Cell cultures were grown in RPMI supplemented with 10% FBS, 2% of glutamine and 1% HEPES (all from Sigma-Aldrich, Steinheim, Germany). As demonstrated by Bartolome et al.[47] and Munoz et al. [48], immortalized lymphoblast cell lines retained the cellular response of freshly cells.

### Western blot analysis

Cells were lysed in buffer containing 10 mM Tris, pH 7.6; 140 mM NaCl; and 0.5 % NP40 including protease inhibitors. After incubation for 20 min on ice, cell debris was cleared by centrifugation. Protein content was determined by a conventional method (BCA protein assay Kit, Pierce, Rockford, IL). Thirty micrograms of protein extracts were electrophoresed separated on 12% SDS-PAGE, and transferred to nitrocellulose membrane (Amersham-GE Life Sciences Healthcare, Milan, Italy). Filters were incubated at room temperature overnight with primary antibodies in 5% non-fat dried milk (Euroclone CELBIO, Milan, Italy). The antibodies used for this study were: antibodies that recognize the two isoforms of Superoxide Dismutase, Cu-Zn Superoxide Dismutase (anti-SOD1, 1∶400 dilution, Santa Cruz Biotechnology Inc., Heidelberg, Germany), and Mn-Superoxide Dismutase (anti-SOD2, 1∶300 dilution, Sigma-Aldrich, St Louis, MO, USA), and anti-α-tubulin antibody (1∶1.500 dilution, Sigma-Aldrich, St Louis, MO, USA). The secondary antibodies (Dako, Glostrup, Denmark) and a chemiluminescence blotting substrate kit (Amersham-GE Life Sciences Healthcare, Milan, Italy) were used for immunodetection. Evaluation of immunoreactivity was performed on immunoblots by densitometric analysis using Scion Image (PC version of Macintosh-compatible NIH Image) software.

### Measurement of protein carbonyls

Briefly, samples (5 µg of proteins) were derivatized with 10 µl 10 mM 2,4-dinitrophenylhydrazine (DNPH) (from OxyBlot^tm^ Protein oxidation Detection Kit, Chemicon-Millipore, Billerica, MA, USA), in the presence of 5 µl of 12% sodium dodecyl sulfate for 20 min at room temperature (23°C). The samples were then neutralized with 7.5 µl of the neutralization solution (2 M Tris in 30% glycerol). Derivatized protein samples were then blotted onto a nitrocellulose membrane with a dot-blot apparatus. The membrane was blocked with a solution of 5% non-fat dried milk in Tris-buffered saline (TBS) solution, and followed by incubation with rabbit polyclonal anti-DNPH antibody (1∶100 dilution, from OxyBlot^tm^ Protein oxidation Detection Kit, Chemicon-Millipore, Billerica, MA, USA) as the primary antibody for 1 h at room temperature. After washing the membrane with TBS buffer, it was further incubated with HRP-conjugated goat anti-rabbit antibody (1∶300 dilution, from OxyBlot^tm^ Protein oxidation Detection Kit, Chemicon-Millipore, Billerica, MA, USA) as the secondary antibody for 1 h at room temperature. Blots were developed using chemiluminescence blotting substrate kit (Amersham-GE Life Sciences Healthcare, Milan, Italy), scanned with Adobe Photoshop, and quantified using Scion Image (PC version of Macintosh-compatible NIH Image) software. Non-specific binding of the primary or secondary antibodies was found.

### Measurement of 3-nitrotyrosine (3-NT)

The Nitro-Tyrosine content was determined immunochemically as previously described [62]. Briefly, samples were incubated with Laemmli sample buffer in a 1∶2 ratio (0.125 M Trizma base, pH 6.8, 4% sodium dodecyl sulfate, 20% glycerol) for 20 min. Protein (5 µg) was then blotted onto the nitrocellulose paper using the slot-blot apparatus and immunochemical methods as described above for protein carbonyls. The primary anti-3NT antibody (Sigma-Aldrich, St Louis, MO, USA) diluted 1∶1000, was incubated at 4°C over night, followed by 3 hours at room temperature and then HRP-conjugated goat anti-rabbit antibody (1∶1500 dilution, Dako, Glostrup, Denmark) at room temperature for 2 hours was used for detection. Blots were then scanned by Adobe Photoshop program, and densitometric analysis of bands in images of the blots was used to calculate levels of 3-NT.

### Measurement of 4-hydroxynonenal (HNE)

Assay was performed as previously described [63]. Briefly, 10 µl of sample were incubated with 10 µl of Laemmli buffer containing 0.125 M Tris base pH 6.8, 4% (v/v) SDS, and 20% (v/v) glycerol. The resulting sample (5 µg) was loaded per well in the slot blot apparatus containing a nitrocellulose membrane under vacuum pressure. The membrane was blocked with a solution of 5% non-fat dried milk in Tris-buffered saline (TBS) solution and incubated with a 1∶200 dilution of anti-HNE polyclonal antibody (Alpha Diagnostic International Inc., San Antonio, Texas 78244 USA) at 4°C over night and 3 hours at room temperature. An anti-rabbit IgG alkaline phosphatase secondary antibody (Dako, Glostrup, Denmark) was diluted 1∶1500 in a solution of 5% non-fat dried milk in Tris-buffered saline (TBS) solution buffer and added to the membrane for 2 hours at room temperature. Quantitative analysis was performed with Scion Image.

### Antioxidant enzyme activity

SOD activity was performed using a buffer (G buffer) that contained 0.05 M glycine (Sigma-Aldrich, St Louis, MO, USA, 0.1 M NaOH (Sigma-Aldrich, St Louis, MO, USA) and 0.1 M NaCl (Sigma-Aldrich, St Louis, MO, USA), pH 10.3, and epinephrine. (Sigma-Aldrich, St Louis, MO, USA).The reaction was monitored in a 96-well plate reader by measuring the decrease of absorbance at 480 nm.

GPx activity was measured by using a reaction mixture consisting of 0.2 mM H_2_O_2_, 1.0 mM GSH, 0.14 U of glutathione reductase (GRD), 1.5 mM NADPH, 1.0 mM sodium azide, and 0.1 M phosphate buffer (pH 7.4) and 1 mg/ml of supernatant protein [64]. The changes in absorbance were recorded at 340 nm in a 96-well microtiter plate.

GRD activity was carried out by using a reaction mixture consisting of 0.1 M phosphate buffer (pH 7.6), 0.5 mM EDTA, 1.0 mM oxidized glutathione, 0.1 mM NADPH, and 10 µl PMS in a total volume of 200 µl [65]. The enzyme activity was assayed in a 96-well plate reader by measuring the disappearance of NADPH at 340 nm.

### Measurement of ROS/RNS generation

Intracellular ROS levels were measured using the fluorescent dye 2′,7′-dichlorodihydrofluorescein diacetate (H_2_DCF-DA) (Molecular Probes), a nonpolar compound that is converted into a nonfluorescent polar derivative (H_2_DCF) by cellular esterases after incorporation into cells. H_2_DCF is membrane permeable and is rapidly oxidized to the highly fluorescent 2′,7′-DCF (**DCF**) in the presence of intracellular ROS [66]. For the experiments, B lymphocytes were washed in Hanks' Balanced Salt Solution (HBSS, GIBCO) and then incubated in nitrogen-saturated HBSS with 20 µM H_2_DCF-DA dissolved in DMSO for 30 minutes at 37°C 5%CO_2_. After washing twice in HBSS, intracellular DCF fluorescence, proportional to the amount of ROS/RNS, was evaluated by flow cytometry.

### Analysis of p53 conformational state

p53 conformational state was analysed by immunopreciptation experiment, and Enzyme-linked Immunosorbent assay (ELISA) assay, using the anti-p53 antibodies, PAb 1620 and PAb 240. In particular, cells were lysed in immunoprecipitation buffer (10 mM Tris, pH 7.6; 140 mM NaCl; and 0.5% NP40 including protease inhibitors) for 20 min on ice, and cell debris was cleared by centrifugation. Protein content was determined by a conventional method (BCA protein assay Kit, Pierce, Rockford, IL). Before immunoprecipitation experiments, an aliquot of 10 µg of protein extracts from each individual sample were processed for Western blot analysis and probed with anti-β tubulin antibody to validate protein content measurements (data not shown). Based on the previous results, one hundred micrograms of protein extracts were used for immunoprecipitation experiments, performed in a volume of 500 µl. To prevent non-specific binding, the supernatant of immunoprecipitated samples was pre-cleared with 10% (w/v) protein A/G (50 µl) (Santa Cruz Biotechnology Inc., Heidelberg, Germany) for 20 min on ice, followed by centrifugation. For immunoprecipitation of p53, 1 µg of the conformation-specific antibodies PAb1620 (wild-type specific) (Calbiochem, EMB Bioscience, La Jolla, CA, USA), PAb240 (mutant specific) (Neomarkers-Lab Vision, Fremont, CA, USA) or PAbBP53.12 (Neomarkers-Lab Vision, Fremont, CA, USA) (recognizing both wild-type and mutant p53) were added to the samples and incubated overnight at 4°C. Immuno complexes were collected by using protein A/G suspension and washed five times with immunopecipitation buffer. Immunoprecipitated p53 was recovered by resuspending the pellets in Laemmli sample buffer. Western blot analysis was performed using as primary antibodies: polyclonal anti-p53 antibody CM1 (Novocastra, Newcastle, UK), polyclonal anti-p53 antibody R19 (Santa Cruz Biotechnology Inc., Heidelberg, Germany), polyclonal anti-HNE and polyclonal anti-3NT antibodies.

For ELISA assay 70 µg of non-denaturated protein extracts were diluted in PBS 1× pH 7.4 and coated on the ELISA microplate overnight at 4°C. The next day plates were saturated with 100 µl of Blocking Solution (PBS 1× pH 7.4–0,1% TWEEN 20 – 3% BSA) for each well and incubated for 1 h at RT, followed by 2 h incubated at 37°C with anti-p53 Pab 240 antibody (0.5 µg/ml). After washing with PBST, 0.1 mg/ml secondary anti-mouse antibody conjugated with peroxidase was incubated in each well for 1 h at RT. Finally, 100 µl of TMB (3,3,5,5-tetramethylbenzidine) substrate is added and the reaction is stopped with 100 µl of sulphuric acid 2 M. Optical density (OD) is measured using a microplate reader with a wavelength of 450 nm. Data was expressed as the mean ± SE of at least three experiments, performed in triplicate.

### Transactivation assay

Transient transfections were performed in 6 multi well culture plates; for each well 7×10^5^ cells were seeded in medium without FBS, antibiotics and with 1% L-glutamine. For transactivation assay cells from control, SAD and FAD subjects were transfected with the p53-target promoter AIP1-luciferase reporter plasmid (kindly provided by H. Arakawa, National Cancer Center, Tokyo, Japan), by using the transfection reagent LTX (Invitrogen Carlsbad, CA) according to the manufacturer's instructions. AIP1-luciferase reporter construct plasmid DNA was co-transfected with pRL-TK Renilla luciferase expressing vector to measure transfection efficiency (Promega, Madison, WI). Eighteen hours later the cells were incubated with 50 µM doxorubicin for 24 h before luciferase activity was assayed. The cells were then lysed with Passive Lysis Buffer 1× provided by Dual-Luciferase Reporter Assay System following the manufacturer's specifications (Promega, Madison, WI). Luminescence was measured employing a 20/20n Luminometer with 10 sec of integration (Turner BioSystems, Sunnyvale, CA). At least six independent experiments were performed.

### Statistical evaluation

Results are given as mean ± standard error mean values. Statistical significance of differences was determined by mean values of the one way ANOVA, followed by the Bonferroni test. Bartlett's test was also performed. Significance was accepted for a p<0.05.
